# Evaluation of electronic order set implementation in patients with diabetic ketoacidosis: a retrospective cohort study

**DOI:** 10.1177/20420188261460595

**Published:** 2026-07-20

**Authors:** Hassan Mitwally, Eman Al Hmoud, Zeana AlKudsi, Mohamed Saad, Farah Zahrah, Mustafa Seidahmed, Mahmud Ben Masoud, Rasha Al-Anany

**Affiliations:** Pharmacy Department, Al-Wakra Hospital, Hamad Medical Corporation, Doha, Qatar; Pharmacy Department, Al-Wakra Hospital, Hamad Medical Corporation, Doha, Qatar; Pharmacy Department, Al-Wakra Hospital, Hamad Medical Corporation, P.O. Box: 82228, Al Wakra, Doha, Qatar; Pharmacy Department, Al-Wakra Hospital, Hamad Medical Corporation, Doha, Qatar; Pharmacy Department, Al-Wakra Hospital, Hamad Medical Corporation, Doha, Qatar; Internal Medicine Department, Al-Wakra Hospital, Hamad Medical Corporation, Doha, Qatar; Critical Care Department, Al-Wakra Hospital, Hamad Medical Corporation, Doha, Qatar; Pharmacy Department, Al-Wakra Hospital, Hamad Medical Corporation, Doha, Qatar

**Keywords:** diabetic ketoacidosis, electronic health records integration, electronic order sets, PowerPlan, resolution, treatment protocol compliance

## Abstract

**Background:**

Diabetic ketoacidosis (DKA) is a life-threatening complication of diabetes requiring timely insulin administration and dynamic fluid/electrolyte management.

**Objectives:**

The aim of this study is to assess the effectiveness of an electronic order set (PowerPlan) on time to DKA resolution compared to individual order prescribing.

**Design:**

Retrospective cohort study.

**Methods:**

This study included patients (⩾14 years old) with DKA, identified via electronic medical records, and matched to pre- and post-PowerPlan groups. The primary outcome was time to DKA resolution. Secondary outcomes included hospital length of stay, appropriateness of initial intravenous fluids, and insulin infusion. Safety outcomes included the number of hypoglycemia, hyperkalemia, and hypokalemia events until DKA resolution.

**Results:**

Of 226 matched patients (113 per group), baseline characteristics were comparable. The post-implementation group achieved faster DKA resolution (median 10.6 vs 12.6 h; *p* = 0.002), higher adherence to fluid and insulin protocols, and shorter hospital stay (3.15 vs 3.61 days; *p* = 0.04). Safety outcomes were similar, with fewer hypoglycemic events and comparable electrolyte abnormalities.

**Conclusion:**

Implementation of the DKA PowerPlan significantly shortened time to resolution and reduced hospital stay without compromising safety. These findings support standardized electronic order sets to enhance efficiency and quality of DKA management.

## Introduction

Diabetic ketoacidosis (DKA) is a life-threatening metabolic complication of type 1 and type 2 diabetes mellitus. It requires prompt and effective management to prevent serious complications, such as severe dehydration, acute kidney injury, and cardiac arrhythmias, in addition to avoiding lengthy and costly hospitalizations.^1,2^ Despite advancements in diabetes management, DKA remains a significant cause of morbidity and mortality, particularly in developing countries.^2^ DKA is characterized by hyperglycemia (>250 mg/dL or >13.9 mmol/L), ketonemia, and metabolic acidosis (arterial pH <7.3 and/or serum bicarbonate <15 mEq/L).^3^ The management of DKA requires a multidisciplinary approach that involves restoration of volume deficits, resolution of hyperglycemia and ketosis, and correction of electrolyte imbalance and acidosis. It is also essential to identify and treat any precipitating event, such as sepsis, myocardial infarction, or stroke.^1,3^ Evidence-based recommendations for managing DKA have been issued by multiple organizations, including the American Diabetes Association (ADA) and the American Association of Clinical Endocrinology. Yet, the availability of clinical guidelines alone does not necessarily translate into optimal care, as variations and delays in treatment practices may still occur. This highlights the importance of implementing a standardized protocol supported by a structured order set to guide timely and effective treatment.

Standardized order sets group medical orders that guide healthcare providers following pre-established evidence-based clinical guidelines. Order sets ensure that all necessary interventions are administered promptly and correctly. It also improves compliance with treatment and reduces variability in care.^4–6^ In addition, order sets have been shown to reduce healthcare resource utilization, including supplies, medications, provider time, and costs.^7^ Several studies have demonstrated the benefits of implementing both standard (paper-based) and computer-based algorithms for the management of DKA.^6,8–15^ Despite these findings, the effectiveness of order sets in improving the management of DKA may depend on various factors, including the design and structure of the order set, the clinical guidelines on which it is based, and the level of acceptance and adherence among healthcare providers.^8,10^

In our hospital, DKA management follows individualized, physician-directed orders. Such a non-standardized approach may increase the likelihood of variation in practice, medication errors, and suboptimal treatment outcomes. Therefore, in this study, we aimed to compare the implementation of the DKA electronic order set at Al-Wakra Hospital (AWH) to the individual orders approach.

## Methodology

This retrospective observational study adheres to the Strengthening the Reporting of Observational Studies in Epidemiology (STROBE) guidelines.^16^ The completed STROBE checklist is provided as a Supplemental Material.

### Study design, setting, and population

This was a single-center retrospective cohort study conducted at AWH, Qatar, one of the facilities under Hamad Medical Corporation (HMC). The study protocol was approved by the Corporate Medical Research Center (MRC-01-23-230), and the requirement for written informed consent was waived due to the retrospective nature of the study.

All patients aged 14 years and older who met the diagnostic criteria for DKA (Table 1)^3^ and were admitted to AWH between January 2016 and August 2023 were eligible for inclusion. The electronic health record (EHR) system (Cerner^®^) was introduced across HMC facilities in 2016. The PRE group included patients diagnosed with DKA prior to protocol implementation, from the introduction of Cerner in 2016 through January 2021. The POST group included patients who had an active DKA electronic order set from September 2021 to August 2023, during which the DKA order set was activated electronically through Cerner by the prescriber at the time of diagnosis.

**Table 1.** ADA diagnostic criteria and classification of DKA.^3^

P﻿arameters	Mild DKA	Moderate DKA	Severe DKA
Plasma glucose (mg/dL)	>250	>250	>250
Arterial pH	7.25–7.30	7.00 to <7.24	<7.00
Serum bicarbonate (mEq/L)	15–18	10 to <15	<10
Serum ketone	Positive	Positive	Positive
Anion gap	>10	>12	>12
Mental status	Alert	Alert/drowsy	Stupor/coma

ADA, American Diabetes Association; DKA, diabetic ketoacidosis.

A washout period from end of January 2021 to end of August 2021 was applied to account for the initial implementation phase of the PowerPlan, during which staff training, workflow adaptation, and gradual uptake of the order set occurred. During this period, ongoing feedback from providers was incorporated to refine and optimize the protocol. Patients admitted during the washout period were excluded to minimize variability associated with partial and evolving adoption. In addition, patients younger than 14 years and those with hyperosmolar hyperglycemic state (HHS) were excluded from the analysis.

### Diabetic ketoacidosis order set (PowerPlan) integration

The hospital implemented an electronic DKA PowerPlan in January 2021. The PowerPlan was developed by a multidisciplinary team and approved by the Corporate Diabetes Committee, aligning with both local (derived from the 2009 ADA guideline) and international DKA management guidelines.^3^ It incorporated standardized order sets for intravenous (IV) fluids, potassium, and insulin (Table 2), which functioned as embedded clinical decision support (CDS) tools by providing pre-configured, guideline-based orders, weight-based dosing guidance, laboratory monitoring orders, and nursing instructions within the order comments to support consistent bedside practice. Once initiated, these electronic orders were automatically transmitted to smart infusion pumps.

**Table 2.** Diabetic ketoacidosis PowerPlan order sentences as integrated in Cerner^®^.

Intravenous fluids and potassium	Dosage forms	Dose/rate	Frequency	Comments
0.9% normal saline	IV bolus	1–2 L	Once	Based on degree of dehydration
Potassium <3.3 mEq/L (Don’t start insulin)				
Potassium chloride 40 mmol in 1000 mL 0.9% normal saline	IV infusion	250 mL/h	Once	Recheck potassium hourly until potassium is ⩾3.3 mEq/L
If serum potassium is 3.3–3.9 mEq/L				
Potassium chloride 40 mmol in 1000 mL 0.45% saline	IV infusion	250 mL/h		If blood sugar 250 mg/dL or more in DKA and 300 mg/dL or more in HHS
Potassium chloride 40 mmol in 1000 mL dextrose 5%/0.45% saline	IV infusion	250 mL/h		If blood sugar 250 mg/dL or less in DKA and 300 mg/dL or less in HHS
If serum potassium is 4–5.1 mEq/L				
Potassium chloride 20 mmol in 1000 mL 0.45% saline	IV infusion	250 mL/h		If blood sugar is 250 mg/dL or more in DKA and 300 mg/dL or more in HHS
Potassium chloride 20 mmol in 1000 mL dextrose 5%/0.45% saline	IV infusion	250 mL/h		If blood sugar is 250 mg/dL or less in DKA and 300 mg/dL or less in HHS
If serum potassium is >5.2 mEq/L				
0.45% saline 1000 mL	IV infusion	250 mL/h		If blood sugar is 250 mg/dL or more in DKA and 300 mg/dL or more in HHS
Dextrose 5% with 0.45% saline 1000 mL	IV infusion	250 mL/h		If blood sugar is 250 mg/dL or less in DKA and 300 mg/dL or less in HHS
**Insulin therapy**				
Insulin regular	IV bolus	0.1 units/kg	Once	
Insulin regular 50 units + sodium chloride 0.9% 50 mL	IV infusion	0.1 unit/kg/h		Consider reducing infusion rate to 0.05 units/kg/h if blood sugar <200 mg/dL, and holding insulin infusion if blood sugar <70 mg/dL
If blood sugar is not reduced by 10% in the 1st hour				
Insulin regular	IV bolus	0.14 units/kg	Once	If blood sugar is not reduced by 10% in the 1st hour
For hypoglycemia^a^				Blood glucose monitoring q1h during insulin infusion
Dextrose 50% in water	IV bolus	25 g	q6h for hypoglycemia	
If pH <6.9				
Sodium bicarbonate 75 mmol in 500 mL dextrose 5%	IV infusion	250 ml/h	Once	

a Hypoglycemia is defined in our institution if blood sugar <69 mg/dL (<3.9 mmol/L).

DKA, diabetic ketoacidosis; HHS, hyperosmolar hyperglycemic state; IV, intravenous; PFS, prefilled syringe; q1h, every 1 h.

Smart infusion pumps are computerized systems that deliver IV medications and fluids with high precision. They utilize dose error-reduction systems and institution-specific drug libraries that verify programmed doses, rates, and concentrations against predefined limits to minimize risks such as overdosing or underdosing.^17^ In our hospital, the smart infusion pumps are integrated with the Cerner^®^ electronic medical record (EMR) system, enabling auto-programming of infusion parameters from verified physician orders and auto-documentation of infusion data back into the patient’s chart, thereby reducing manual entry errors and improving infusion safety.

Prior to implementation, structured educational sessions were conducted for physicians and nurses working in the Emergency Department, intensive care unit (ICU), and internal medicine wards. These sessions included presentations outlining the DKA protocol and step-by-step walkthroughs of the electronic PowerPlan. Moreover, simulation exercises were used to familiarize staff with workflow and decision-making using the order set. Nursing educators and clinical pharmacists delivered the training and provided on-site support during the early implementation phase to reinforce adoption and address questions in real time. To support sustained adherence, periodic refresher sessions and orientation training for newly rotating staff were conducted, and pharmacists and nurse educators remained available to provide ongoing guidance.

### Data collection

Data were extracted retrospectively from the EMR of eligible patients. Baseline data were obtained at the time of hospital admission and included demographic information (gender, age, and weight), history of diabetes (Type 1, Type 2, or new diagnosis), home insulin use, medication history, and relevant comorbidities such as hypertension, heart failure (HF), coronary artery disease (CAD), and chronic kidney disease (CKD). DKA-specific variables, including initial laboratory values, severity of presentation, and the number of prior ICU admissions for DKA, were also collected.

### Outcomes

The primary outcome of this study was to assess the impact of the PowerPlan on the time to DKA resolution, measured in hours. Time to DKA resolution was defined as the duration from the time of DKA diagnosis (Table 1) to the time at which resolution criteria were met. Resolution of DKA was defined by the presence of at least two of the following criteria: serum bicarbonate level ⩾15 mEq/L, venous pH >7.3, and anion gap closure ⩽12 mEq/L.^3^ Secondary outcomes included comparisons between the PRE- and POST-implementation groups in terms of hospital length of stay (LOS) for non-ICU patients, ICU LOS, appropriate initiation of maintenance IV fluids, and appropriate initiation of IV insulin infusion. In addition, DKA recurrence and mortality were assessed. Compliance with the full PowerPlan order set was evaluated for the POST group only.

Initial maintenance fluid appropriateness was assessed by calculating the corrected sodium using Katz’s equation (1973) and determining whether the selected fluid was appropriate for the sodium level. For corrected sodium between 130 and 135 mmol/L, either normal saline (NS) or half-NS (0.45% NS) was considered appropriate; if above 135 mmol/L, 0.45% NS was recommended; and if below 130 mmol/L, NS was appropriate. Once the plasma glucose is <13.9 mmol/L (<250 mg/dL), 5% dextrose (D5) should be added to replacement fluids. The maintenance fluid rate was typically initiated at 250 mL/h in accordance with institutional protocol and guideline recommendations for patients without cardiac or renal compromise; however, further rate adjustments were individualized based on clinical assessment, comorbidities (e.g., HF or CKD), fluid balance monitoring, and laboratory parameters.^3^

Insulin therapy was considered appropriate when initiated at a fixed weight-based continuous IV infusion of 0.1 units/kg/h, in accordance with DKA management guidelines.^3^ Blood glucose levels were monitored every hour during insulin infusion to guide clinical management and ensure safe glycemic control.

Safety outcomes evaluated were the number of hypoglycemic events (defined as blood glucose <3.89 mmol/L or <70 mg/dL) during insulin infusion, as well as the number of hyperkalemia events (serum potassium >5.3 mEq/L) and hypokalemia events (serum potassium <3.3 mEq/L) observed until DKA resolution. Electrolyte disturbances were assessed as binary safety outcomes (presence or absence) based on institutional laboratory thresholds, and severity ranges were not further stratified.

### Statistical consideration and data analysis

Categorical data were summarized and expressed as frequency with percentage. Numeric data were summarized and expressed as mean and standard deviation if normally distributed, or median with interquartile range (IQR) if not normally distributed. Patients in the post-implementation period were matched to patients in the pre-implementation period based on age (10-year interval) and severity of DKA. Demographics and baseline characteristics were compared using McNemar’s test for categorical variables, paired *t*-test for normally distributed continuous variables, and Wilcoxon signed-rank test for non-normally distributed continuous or ordinal variables. Time to resolution of DKA and LOS (ICU and hospital) outcomes were compared using the Wilcoxon signed-rank test. Appropriate selection of IV fluids and insulin infusion, as well as safety outcomes, were assessed using McNemar’s test. Two-sided *p* values <0.05 were considered statistically significant. Statistical analyses were performed using Stata version 17.

## Results

A total of 447 patients were identified, of whom 334 patients were in the pre-implementation period and 113 patients were in the post-implementation period. After matching, 226 patients were included in the final analysis, with 113 in the pre-implementation group (PRE group) and 113 in the post-implementation group (POST group). Baseline demographics and clinical characteristics are presented in Table 3. There were no statistically significant differences between groups in gender distribution (*p* = 0.069), median age (32 vs 34 years; *p* = 0.56), ICU admission rates (*p* = 0.13), or initial laboratory values, including pH, anion gap, bicarbonate, and glucose levels. The proportion of patients with known type 1 or type 2 diabetes, newly diagnosed diabetes, or pre-admission insulin use did not differ significantly between groups. Comorbidities such as hypertension, CAD, HF, and CKD were also comparable between groups. Due to matching, the severity of DKA was identical across both groups, with 10.6% classified as mild, 43.4% as moderate, and 46.0% as severe.

**Table 3.** Comparison of baseline patients’ characteristics between PRE and POST groups.

Characteristic	PRE group	POST group	*p*-Value
	*n* = 113	*n* = 113	
Female	30 (26.6%)	43 (38.1%)	0.07
Age (years) (median, IQR)	32 (22–45)	34 (24–44)	0.56
ICU admission	44 (38.9%)	39 (34.5%)	0.13
*Laboratory tests*			
pH (median, IQR)	7.16 (7.07–7.22)	7.17 (7.09–7.23)	0.07
Anion gap (median, IQR)	21.5 (16.8–25.3)	21.2 (17–24.5)	0.94
Bicarbonate (mEq/L) (median, IQR)	10.5 (6.4–13)	10.7 (6.8–14.2)	0.31
Glucose (mmol/L) (median, IQR)	23.3 (18.7–29)	21.2 (17.6–26)	0.09
Known DM Type I	42 (37.2%)	41 (36.3%)	
Known DM Type II	45 (39.8%)	52 (46.0%)	
Newly diagnosed DM	26 (23%)	20 (17.7%)	0.30
On insulin before admission	73 (64.6%)	65 (57.5%)	0.25
Hypertension	15 (13.3%)	17 (15.0%)	0.81
CAD	6 (5.3%)	1 (0.9%)	0.13
Heart failure	1 (0.9%)	0 (0.0%)	>0.99
CKD	6 (5.3%)	4 (3.5%)	0.75

Median (IQR), or *N* (%).

CAD, coronary artery disease; CKD, chronic kidney disease; DM, diabetes mellitus; ICU, intensive care unit; IQR, interquartile range.

The study outcomes are summarized in Table 4. While DKA resolution was achieved in nearly all patients in both groups, the time to resolution was significantly shorter in the POST group (median 10.6 h (IQR: 6.8–16.4)) compared to the PRE group (12.6 h (IQR: 7.9–20.7); *p* = 0.002). Moreover, a significantly higher proportion of patients in the POST group received appropriate first maintenance fluid compared to the PRE group (83.2% vs 55.8%; *p* < 0.001). Similarly, the rate of correct insulin infusion prescription was significantly higher in the POST group (96.5%) compared to the PRE group (23.0%; *p* < 0.001). The hospital LOS was also significantly reduced in the post-implementation group (3.15 vs 3.61 days; *p* = 0.04). There were no significant differences between groups in ICU LOS (*p* = 0.53), DKA recurrence (*p* = 0.55), or hospital mortality (*p* = 0.25). Among the POST group, 69% of patients were managed in full compliance with the new protocol.

**Table 4.** Primary, secondary, and safety outcomes.

Outcomes	PRE group	POST group	*p*-Value
	*n* = 113	*n* = 113	
Time to resolution (h) (median, IQR)	12.63 (7.86–20.68)	10.6 (6.82–16.43)	0.002
DKA resolved	112 (99.1%)	113 (100.0%)	>0.99
DKA recurred	28 (24.8%)	24 (21.2%)	0.55
First maintenance fluid appropriateness	63 (55.8%)	94 (83.2%)	<0.001
Insulin infusion appropriateness	26 (23.0%)	109 (96.5%)	<0.001
Hospital mortality	3 (2.7%)	0 (0.0%)	0.25
*Hospital LOS*			
Hospital LOS (days) (median, IQR)	3.61 (2.66–5.82)	3.15 (2.34–4.65)	0.04
ICU LOS (days) (median, IQR)	2.00 (1.00–3.00)	2.00 (2.00–3.00)	0.53
Compliance with the PP (for POST-group)		78 (69.0%)	
*Safety*			
Hypoglycemia during infusion (BG <69 mg/dL)	15 (13.3%)	7 (6.2%)	0.08
Hypokalemia events (*K* < 3.3 mEq/L)	43 (38.1%)	44 (38.9%)	0.88
Hyperkalemia events (*K* > 5.3 mEq/L)	7 (6.2%)	17 (15.0%)	0.06

Median (IQR) or *N* (%).

BG, blood glucose; DKA, diabetic ketoacidosis; ICU, intensive care unit; IQR, Interquartile range; LOS, length of stay; PP, PowerPlan.

Safety outcomes (Table 4) show a trend toward fewer hypoglycemic events in the POST group (6.2%) compared to the PRE group (13.3%), though this did not reach statistical significance (*p* = 0.08). The incidence of hypokalemia events remained similar between the two groups (*p* = 0.88). Although not statistically significant, there was a higher rate of hyperkalemia in the POST group compared to the PRE group (15.0% vs 6.2%; *p* = 0.064).

## Discussion

In this study, we evaluated the effectiveness of implementing an electronic DKA PowerPlan. To our knowledge, this is one of the first studies to compare a computerized order set and decision support system with conventional protocols in patients admitted to the hospital. Our findings demonstrate that the use of this standardized electronic order set may improve adherence to international treatment protocols, shorten the time to metabolic resolution, and reduce hospital LOS. In particular, the implementation of the Cerner DKA PowerPlan was associated with a significantly shorter time to resolution of DKA compared with the pre-implementation period. In addition, a significantly higher proportion of patients in the POST group received the appropriate first maintenance fluids and a correct weight-based insulin infusion prescription compared with the PRE group. These findings highlight the role of standardized electronic order sets in facilitating the timely and consistent delivery of insulin therapy, fluid resuscitation, and electrolyte replacement.

Similar improvements have been observed in previous studies where order sets and algorithms were evaluated in the management of DKA.^6,8–13,18,19^ Two studies have demonstrated the effectiveness of computerized insulin dosing tools (CIDTs) integrated into the EMR: Defayette et al.^13^ evaluated GlucoStabilizer™ and Ullal et al.^20^ studied Glucommander™. Both CIDTs calculate insulin infusion rates based on real-time blood glucose values and patient-specific variables, allowing automated dose recommendations that aim to optimize glycemic control. In Defayette et al.,^13^ the pre-intervention group lacked a comprehensive, standardized insulin dosing protocol for nurses, resulting in non-standardized management. Similarly, Ullal et al.^20^ compared a paper-based or column-based insulin algorithm with the computerized Glucommander, which was computerized but not fully standardized. In contrast, our study integrates both standardization and computerization by implementing a fully standardized electronic order set that replaced all paper-based protocols, ensuring consistent, guideline-concordant DKA management across all patients.

In comparison to our study, previous studies implemented paper-based algorithms.^6,8,11,14,19^ Hara et al.^19^ and Maghrabi et al.^8^ demonstrated reductions in both time to DKA resolution and time to anion gap closure from 22.8 h in the pre-order set group to 13.6 h in the post-order set group (*p* < 0.001), and from 11.5 h in the pre-order set to 8.5 h in the post-order set (*p* = 0.008), respectively. However, Hara et al. reported that patients in the non-protocol group presented with significantly more severe illness, as reflected by use of mechanical ventilation, lower pH, and higher rates of septic shock. Whereas our study’s population consisted of well-balanced groups in terms of severity of DKA, ICU admission rate, baseline pH, and comorbidities, allowing for a more balanced comparison of the impact of the electronic order set. In addition, Hara et al. included a greater proportion of mixed DKA and HHS cases, which may have influenced the outcomes. Although the authors conducted a subgroup analysis to address this limitation, the differences between groups were not statistically significant for the DKA-only population.^19^

Furthermore, Clark et al.^6^ and Mohamed et al.,^11^ evaluated the implementation of a paper-based DKA management form based on the Diabetes Canada 2013 Guideline.^21^ Their protocols utilized a variable-rate intravenous insulin infusion (VRIII) approach rather than the fixed-rate intravenous insulin infusion (FRIII) advocated by multiple international guidelines, including those from the ADA and the Joint British Diabetes Societies.^3,22^ The main concern with the VRIII approach is its association with prolonged insulin infusion duration and longer hospital LOS.^23^ By contrast, international guidelines recommend the use of a weight-based fixed insulin infusion rate of 0.1 units/kg/h during the initial phase of treatment, with subsequent rate adjustments once plasma glucose drops below 200 mg/dL.^3^ Our study incorporated this evidence-based FRIII protocol directly into the electronic DKA order set, ensuring standardized insulin management aligned with international recommendations. Moreover, Mohamed et al.^11^ included a relatively small cohort of 110 patients, whereas our study involved a larger sample of 226 patients, providing greater statistical power and generalizability.

Likewise, in a medical ICU setting, Laliberte et al.^18^ found that the use of a computerized DKA order set and protocol resulted in significantly lower median time to DKA resolution in the post-order set compared to the pre-order set group (9 vs 11 h, *p* = 0.04). Despite this study showing similar findings regarding efficacy outcome, the post-computerized DKA order set implementation group showed a significant increase in hypoglycemic events when compared to the pre-group (38% vs 13%, respectively). Notably, our study observed a lower incidence of hypoglycemia in the PowerPlan group (6.2% vs 13%), highlighting the safety of a standardized electronic order set integrated with real-time CDS.

From a safety perspective, there were no significant differences between groups. Although post-implementation hypoglycemia rates were lower (6.2% vs 13.3%), the difference did not reach statistical significance. This trend aligns with prior findings suggesting that protocolized DKA management can help mitigate hypoglycemia risk without increasing electrolyte disturbances.^8–10,19^ A slightly higher, but also non-significant, incidence of hyperkalemia was observed in the post-implementation group. This finding may be explained by the use of the upper recommended potassium concentration (40 mmol/L) in patients with initial serum potassium levels between 3.3 and 3.9 mmol/L. This adjustment was necessary because a 30 mmol/L potassium concentration was unavailable in our smart infusion pump library, making the creation and integration of a new order entry across all pumps operationally challenging.

Adherence to DKA order sets has been reported at 83% in pediatric populations and 84% in adult populations.^6,24^ In comparison, our post-implementation compliance rate with all PowerPlan components was 69%. Although this rate appears lower than those in previous studies, it reflects adherence to the entire PowerPlan rather than partial utilization. Importantly, activation of the full order set was not mandatory, which may partially explain the lower rate. This finding underscores the importance of pairing standardized order sets with clinician education, system integration, and routine quality monitoring to ensure sustained adherence and effectiveness. Beyond DKA, studies evaluating order sets in other acute conditions, such as sepsis and community-acquired pneumonia, have also demonstrated reductions in time to key clinical milestones, further highlighting the value of standardized, guideline-based pathways in improving acute care outcomes and promoting practice consistency.^4,5,7^

The implementation of the PowerPlan alongside staff education and implementation support reflects a comprehensive and pragmatic approach to improving DKA management and facilitating sustained adherence to standardized protocols in real-world clinical practice. These findings suggest that the PowerPlan may be most effective when implemented as part of a bundled strategy with education and training to optimize its use and impact.

This study has numerous strengths. It includes a relatively large sample size (*n* = 226) compared with previously published DKA order set studies,^6,12,14,18,24^ providing real-world data that enhances external validity. Most notably, to our knowledge, this is the first study to implement an electronically integrated standardized clinical guide and algorithm for the complete management of DKA, offering clinicians structured, real-time decision support from presentation through resolution. Previous investigations focused mainly on static or paper-based order sets,^6,11,19^ or standardized written protocols,^8,14,18^ which are limited in adaptability and depend heavily on user interpretation. Our approach addressed these gaps by embedding an interactive algorithm into clinical workflows supporting standardized, timely, and individualized care consistent with recommendations for technology-assisted DKA management.

### Limitations

Our study has several limitations that should be acknowledged. First, its retrospective design inherently limits the ability to establish causal relationships. Second, as a single-center study, the findings may not be fully generalizable to other institutions with different patient EHR systems or clinical workflows. Third, in the PowerPlan group, adherence to the full electronic order set was not mandatory and was only 69%, which could have diluted the observed benefits and obscured the potential efficacy of full implementation. Although DKA recurrence was reported as an outcome, the study did not evaluate the transition protocol from IV to subcutaneous insulin or the subsequent management following resolution, which may influence recurrence rates. In addition, the implementation of the PowerPlan occurred alongside staff education and implementation support, which may have contributed to the observed improvements; therefore, the effects cannot be attributed solely to the PowerPlan and should be interpreted as part of a bundled implementation approach. Finally, a formal sample size calculation was not performed, as all eligible patients during the study period were included; therefore, the study may be underpowered to detect smaller differences in secondary outcomes. Future prospective multicenter studies with larger sample sizes are warranted to validate these findings, assess cost-effectiveness, and further evaluate the impact of standardized electronic algorithms on DKA outcomes.

## Conclusion

This study evaluated the impact of an electronic PowerPlan for DKA management. Our findings provide important insights into how standardized electronic order sets can improve adherence to guideline-based management. Implementing an electronic order set for DKA management was associated with improved adherence to treatment protocols, faster metabolic correction, and shorter hospital stays without increasing adverse safety events. These findings add to existing evidence that structured, guideline-based protocols can enhance the quality and consistency of DKA care in real-world settings. Moreover, this initiative can trigger the use of electronic-based order sets to improve the outcomes of other diseases with complicated or interrelated treatment algorithms. Future research should focus on broader implementation strategies, long-term outcomes, and cost-effectiveness to guide best practices in DKA management.

## Supplemental Material

sj-docx-1-tae-10.1177_20420188261460595 – Supplemental material for Evaluation of electronic order set implementation in patients with diabetic ketoacidosis: a retrospective cohort study Supplemental material, sj-docx-1-tae-10.1177_20420188261460595 for Evaluation of electronic order set implementation in patients with diabetic ketoacidosis: a retrospective cohort study by Hassan Mitwally, Eman Al Hmoud, Zeana AlKudsi, Mohamed Saad, Farah Zahrah, Mustafa Seidahmed, Mahmud Ben Masoud and Rasha Al-Anany in Therapeutic Advances in Endocrinology and Metabolism

## Declarations

**Ethics approval and consent to participate:** This study was approved by the ethical committee, Medical Research Center (MRC), Hamad Medical Corporation, under the number: MRC-01-23-230. This is an IRB-approved retrospective study; all patient information was de-identified, and patient consent was not required. Patient data will not be shared with third parties.

**Consent for publication:** Not applicable.

**Availability of data and materials:** The datasets generated during and/or analyzed during the current study are available from the corresponding author on reasonable request.*
